# Supplementary material for: Assessment of Evolutionary Relationships for Prioritization of Myxobacteria for Natural Product Discovery
Source: Microorganisms. 2021 Jun 24;9(7):1376. doi: 10.3390/microorganisms9071376 (PMC8307915; doi:10.3390/microorganisms9071376)
Supplement: Supplementary file 1 [file microorganisms-09-01376-s001.zip › microorganisms-1187135-supplementary.pdf]

microorganisms

Communication

Assessment of Evolutionary Relationships for Prioritization of Myxobacteria for Natural Product Discovery

Andrew Ahearne[1,#], Hanan Albataineh[1,#], Scot E. Dowd[2], and D. Cole Stevens [1,*]

[1] Department of BioMolecular Sciences, School of Pharmacy, University of Mississippi, University, MS 38677, USA; haalbata@go.olemiss.edu, aahearne@go.olemiss.edu
[2] MR DNA, Molecular Research LP, Shallowater, Texas, USA
* Correspondence: stevens@olemiss.edu; Tel.: +1-662-915-5730

[#] These authors contributed equally.

Received: date; Accepted: date; Published: date

Table S1. Novel myxobacteria described in literature spanning 2011-2021. References to all strains below included in manuscript [1-16]

strain	year described
Myxococcus eversor sp. nov.	2020
Myxococcus llanfairpwllgwyngyllgogerychwyrndrobwllllantysiliogogogochensis sp. nov.	2020
Myxococcus vastator sp. nov.	2020
Pyxidicoccus caerfyrddinensis sp. nov.	2020
Pyxidicoccus trucidator sp. nov.	2020
Corallococcus aberystwythensis sp. nov.	2020
Corallococcus carmarthensis sp. nov.	2020
Corallococcus exercitus sp. nov.	2020
Corallococcus interemptor sp. nov.	2020
Corallococcus llansteffanensis sp. nov.	2020
Corallococcus praedator sp. nov.	2020
Corallococcus sicarius sp. nov.	2020
Corallococcus terminator sp. nov.	2020
Sorangium ambruticinum sp. nov.	2018
Sorangium arenae sp. nov.	2018
Sorangium bulgaricum sp. nov.	2018
Sorangium dawidii sp. nov.	2018
Sorangium kenyense sp. nov.	2018
Sorangium orientale sp. nov.	2018
Sorangium reichenbachii sp. nov.	2018
Simulacricoccus ruber gen. nov., sp. nov.	2018
Nannocystis konarekensis sp. nov.	2018
Vitiosangium cumulatum gen. nov., sp. nov.	2017
Vitiosangium subalbum sp. nov.	2017
Racemicystis persica sp. nov.	2017
Racemicystis crocea gen. nov., sp. nov.	2016
Aetherobacter fasciculatus gen. nov., sp. nov.	2016
Aetherobacter rufus sp. nov.	2016
Myxococcus hansupus	2016
Aggregicoccus edonensis gen. nov., sp. nov.	2015
Minicystis rosea gen. nov., sp. nov.	2014
Vulgatibacter incomptus gen. nov., sp. nov.	2014
Labilithrix luteola gen. nov., sp. nov.	2014
Pseudenhygromyxa salsuginis gen. nov., sp. nov.	2013
Sandaracinus amylolyticus gen. nov., sp. nov.	2012
	**35 novel species total**
	**10 novel genera total**

Table S2. Comparison of biosynthetic gene clusters (BGCs) located on contig edges from previously sequenced members of the genus *Corallococcus***.** Included BGC data provided by whole genome analysis using antiSMASH version 5.

strain	accession #	total BGCs	BGCs on contig edge	average % of BGCs on contig edge
C. coralloides DSM 2259^T	NC_017030.1	34	0	0.00
C. coralloides B035	NZ_CP034669.1	34	0	0.00
C. exgiuus DSM 14696^T	JAAAPK010000001.1	44	13	29.55
C. interemptor AB047A^T	RAWM01000001.1	45	34	75.56
C. praedator CA031B^T	RAWI01000001.1	70	66	94.29
C. aberystwythensis AB050A^T	RAWK01000001.1	46	41	89.13
C. sicarius CA040B^T	RAWG01000001.1	68	61	89.71
C. carmarthensis CA043D^T	RAWE01000001.1	55	46	83.64
C. exercitus AB043B	JABFJV010000005.1	59	52	88.14
C. terminator CA054A^T	RAVZ01000001.1	71	68	95.77
C. llansteffanensis CA051B^T	RAWB01000001.1	95	95	100.00
**total # of BGCs: 621**			**overall average # of BGCs on contig edge: 67.80**	

Table S3. Locations of soil samples used for environmental strains isolation

Myxobacteria	City	Longitude and Latitude
NCSPR001 & NCCRE002	Asheville, NC, USA	35°37'30.3"N 82°32'53.2"W
SCHIC003 & SCPEA002	Landrum, SC, USA	35°08'41.3"N 82°09'48.2"W

List S1. A list of all myxobacteria and their accession numbers used in BiG-SCAPE analysis.

1. *Aggregicoccus* sp.17bor-14 (NZ_VJZZ00000000.1)
2. *Anaeromyxobacter dehalogenans* 2CP-C (NC_007760.1)
3. *Anaeromyxobacter* sp Fw109-5 (NC_009675.1)
4. *Anaeromyxobacter* sp. K (NC_011145.1)
5. *Anaeromyxobacter dehalogenans* 2CP-1 DSM 21875^{T} (NC_011891.1)
6. *Archangium* sp.Cb G35 (NZ_MPOI00000000.1)
7. *Archangium gephyra* DSM 2261^{T} (NZ_CP011509.1)
8. *Archangium violaceum* Cb vi76 (NZ_JPMI00000000.1)
9. *Corallococcus exiguus* DSM 14696^{T} (NZ_JAAAPK000000000.1)
10. *Corallococcus exercitus* AB043B (NZ_JABFJV000000000.1)
11. *Corallococcus carmarthensis* CA046B (JABFJX000000000.1)
12. *Corallococcus macrosporus* HW-1 (NC_015711.1)
13. *Corallococcus coralloides* DSM 2259^{T}(NC_017030.1)
14. *Corallococcus coralloides* B035 (NZ_CP034669.1)
15. *Corallococcus terminator* CA054A DSM 108848^{T} (RAVZ00000000.1)
16. *Corallococcus llansteffanensis* CA051B DSM 108844^{T} (RAWB00000000.1)
17. *Corallococcus sicarius* CA040B DSM 108850^{T} (RAWG00000000.1)
18. *Corallococcus aberystwythensis* AB050A DSM 108846^{T}(RAWK00000000.1)
19. *Corallococcus interemptor* AB047A DSM 108843^{T} (NZ_RAWM00000000.1)
20. *Cystobacter fuscus* DSM 2262^{T} (NZ_ANAH00000000.2)
21. *Cystobacter fuscus* DSM 52655 (NZ_CP022098.1)
22. *Cystobacter ferrugineus* Cbfe23 (NZ_MPIN00000000.1)
23. *Hyalangium minutum* DSM 14724^{T} (NZ_JMCB00000000.1)
24. *Melittangium boletus* DSM 14713^{T} (NZ_CP022163.1)
25. *Myxococcus fulvus* 124B02 (CP006003.1)
26. *Myxococcus fulvus* DSM 16525^{T} (NZ_FOIB00000000.1)
27. *Myxococcus hansupus* mixupus (NZ_CP012109.1)
28. *Myxococcus macrosporus* DSM 14697^{T} (NZ_CP022203.1)
29. *Myxococcus stipitatus* DSM 14675^{T} (NC_020126.1)
30. *Myxococcus xanthus* DK1622 (NC_008095.1)
31. *Myxococcus xanthus* GH3.5.6c2 (NZ_CP017169.1)
32. *Myxococcus xanthus* GH5.1.9c20 (NZ_CP017170.1)
33. *Myxococcus xanthus* KF3.2.8c11 (NZ_CP017171.1)
34. *Myxococcus xanthus* KF4.3.9c1 (NZ_CP017172.1)
35. *Myxococcus xanthus* MC3.3.5c16 (NZ_CP017173.1)
36. *Myxococcus xanthus* MC3.5.9c15 (NZ_CP017174.1)
37. *Myxococcus xanthus* DK1622 (NZ_CP065375.1)
38. *Myxococcus virescens* NBRC 100334 (NZ_BJVY00000000.1)
39. *Myxococcus virescens* DSM 2260^{T} (NZ_FNAJ00000000.1)
40. *Pyxidicoccus fallax* DSM 14698^{T} (JABBJJ000000000.1)
41. *Stigmatella aurantiaca* DSM 17044^{T} (NZ_FOAP00000000.1)
42. *Stigmatella aurantiaca* DW43-1 (NC_014623.1)
43. *Stigmatella erecta* DSM 16858^{T} (NZ_FOIJ00000000.1)
44. *Vitiosangium* sp.GDMCC1.1324 (NZ_PZOX00000000.1)
45. *Vulgatibacter incomptus* DSM 27710 (NZ_CP012332.1)

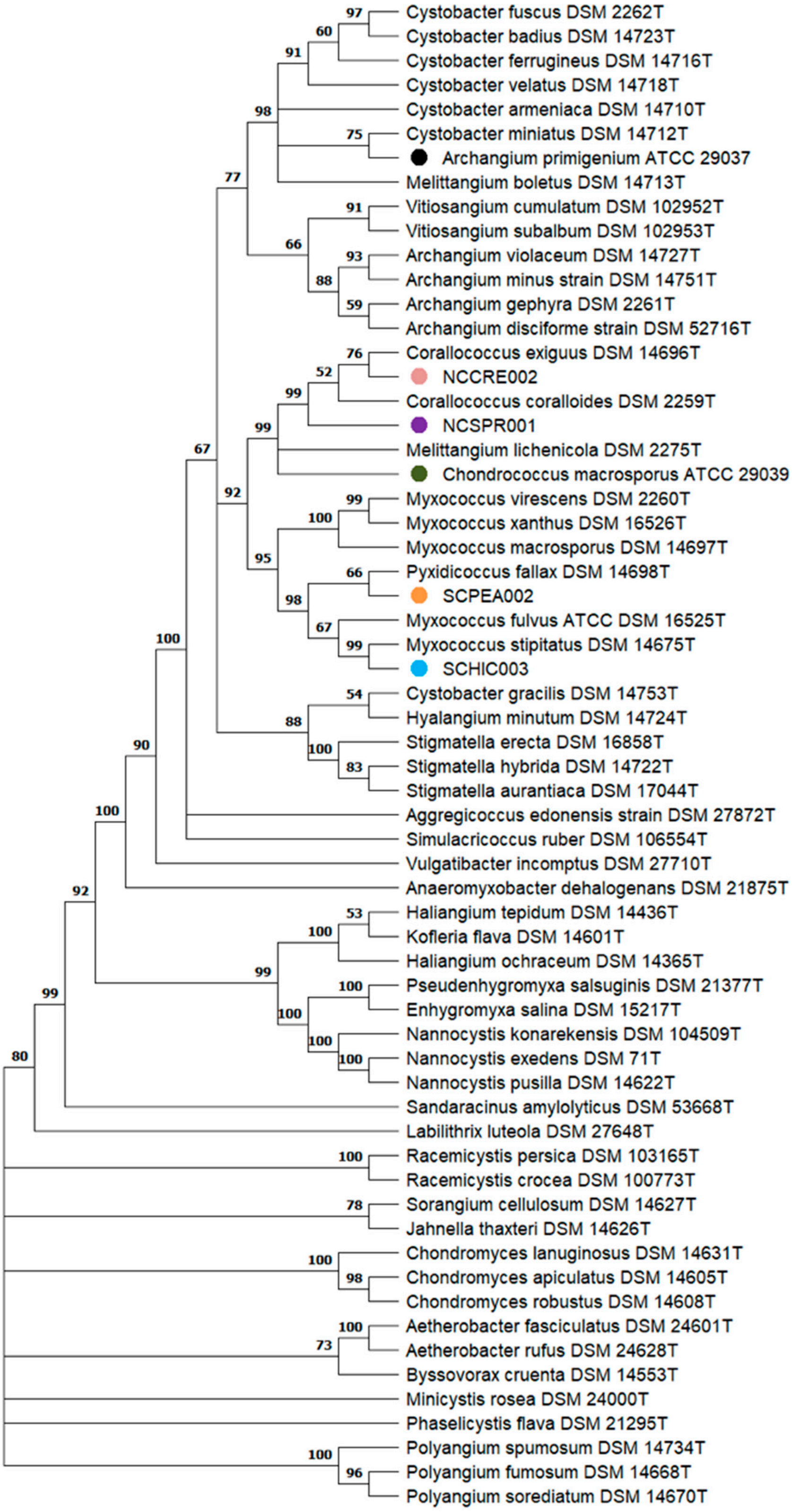

Figure S1. Minimum evolution tree from the 16S rRNA of the 6 strains under investigation in this study and myxobacterial type strains deposited in DSMZ. The letter "T" at the end of strain identifiers indicates type strain. The optimal tree with the sum of branch length = 0.88149497 is shown. The percentage of replicate trees in which the associated taxa clustered together in the bootstrap test (5000 replicates) are shown above the branches in bold. Branch pseudo-bootstrap less than 50% are not shown.

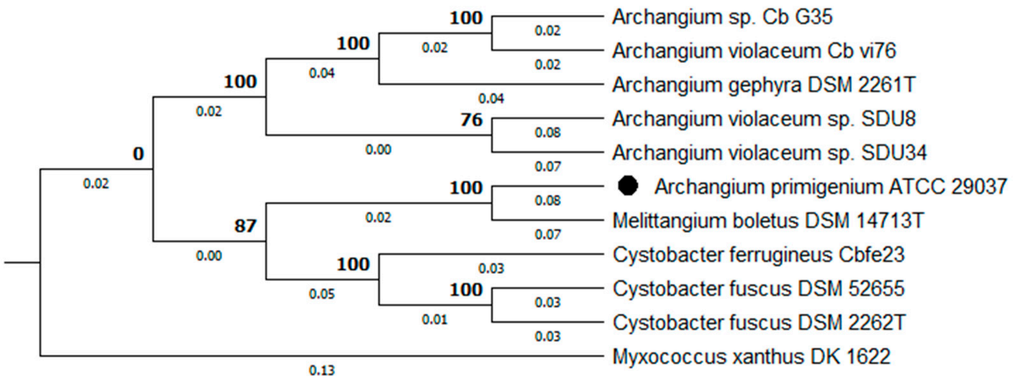

Figure S2. Minimum evolution tree from the whole genomes of *A. primigenium* and different members of the family *Archangiaceae* using the GBDP approach. The letter "T" at the end of strain identifiers indicates type strain. The numbers in bold above the branches are GBDP pseudo-bootstrap support values > 60 % from 100 replications, with an average branch support of 100.0 %. The numbers below branches are branch lengths scaled in terms of GBDP distance formula d5. branch lengths less than 0.05 are not shown. The tree was rooted at the midpoint. *Myxcococcus xanthus* DK 1622 is included in the GBDP tree as an outgroup.

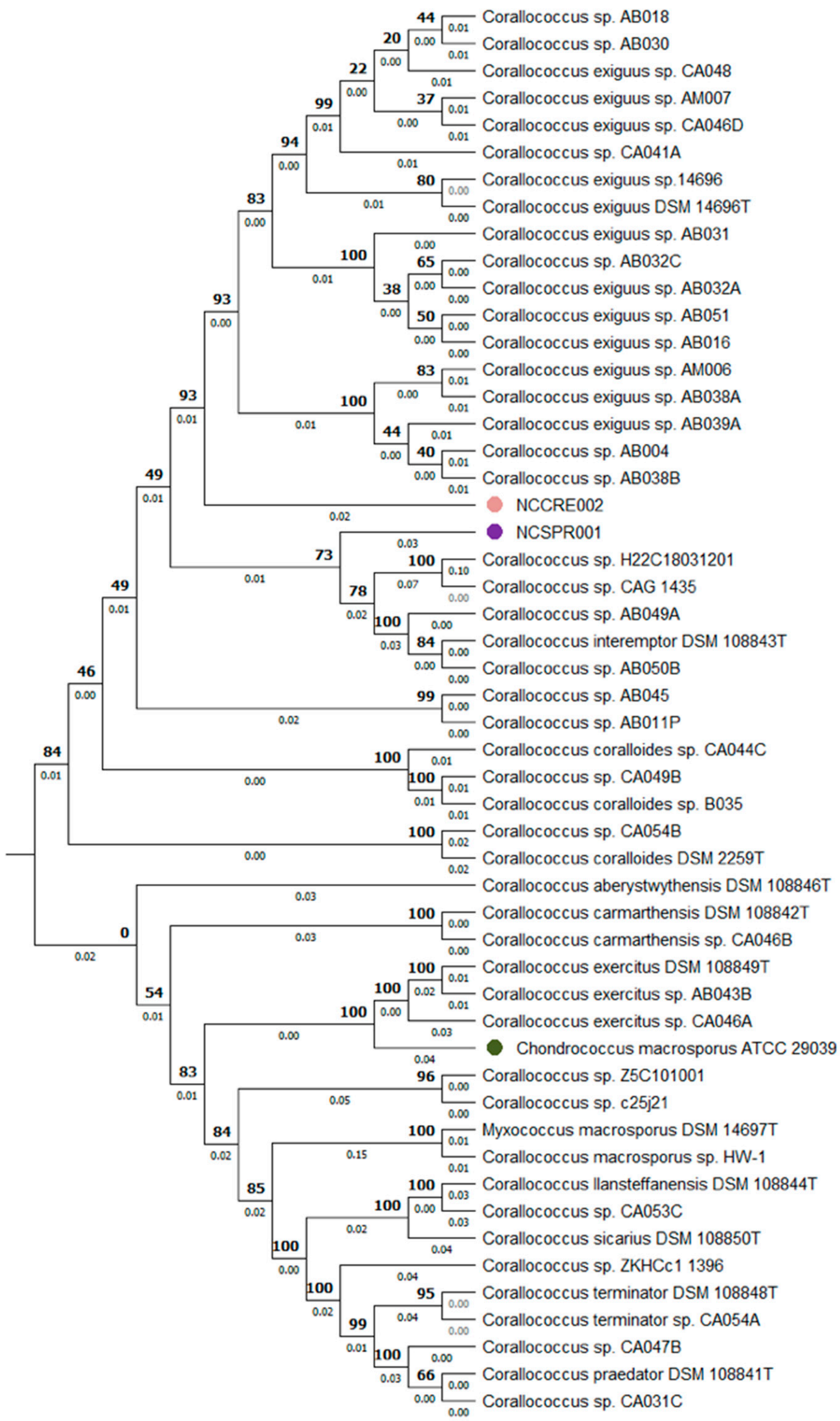

Figure S3. Minimum evolution tree from the whole genomes of *C. macrosporus* ATCC 29039, NCCRE002, NCSPR001 and different members of genus *Corallococcus* using the GBDP approach. The letter "T" at the end of strain identifiers indicates type strain. The numbers in bold above the branches are GBDP pseudo-bootstrap support values > 60 % from 100 replications, with an average branch support of 100.0 %. Branch pseudo-bootstrap less than 50% are not shown. The numbers below branches are branch lengths scaled in terms of GBDP distance formula d5. The tree was rooted at the midpoint.

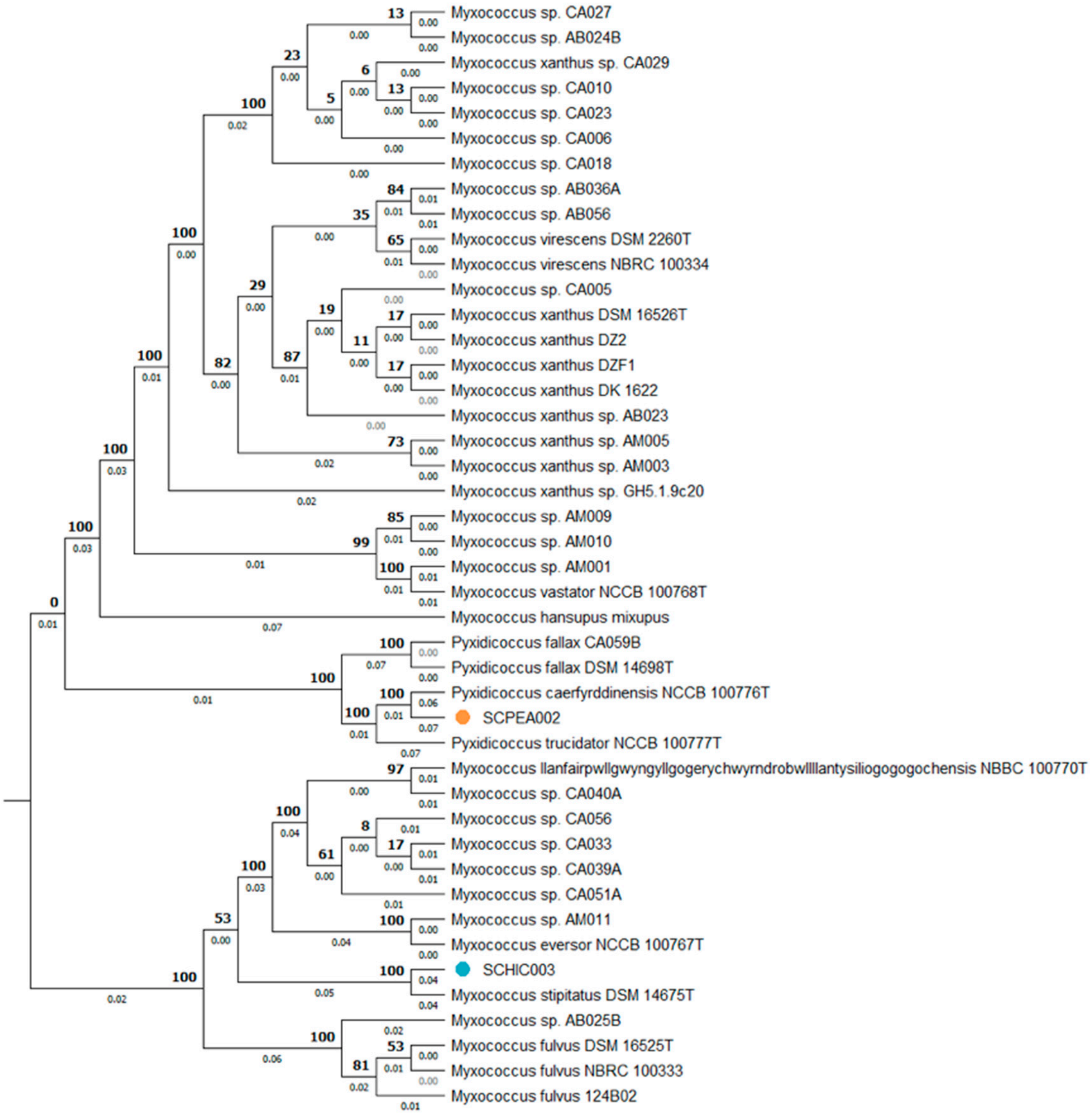

Figure S4. Minimum evolution tree from the whole genomes of SCHIC003, SCPEA002, and different members of *Myxococcus* and *Pyxidicoccus* using the GBDP approach. The letter "T" at the end of strain identifiers indicates type strain. The numbers in bold above the branches are GBDP pseudo-bootstrap support values > 60 % from 100 replications, with an average branch support of 100.0 %. Branch pseudo-bootstrap less than 50% are not shown. The numbers below branches are branch lengths scaled in terms of GBDP distance formula d5. The tree was rooted at the midpoint.
